# Metformin inhibits the proliferation of A549/CDDP cells by activating p38 mitogen-activated protein kinase

**DOI:** 10.3892/ol.2014.2270

**Published:** 2014-06-19

**Authors:** YAJUN WANG, BIYUN LIN, JUN WU, HAITAO ZHANG, BIN WU

**Affiliations:** 1 Institute of Respiratory Diseases, Affiliated Hospital of Guangdong Medical College, Zhanjiang, Guangdong 524023, P.R. China; 2 Department of Biochemistry and Molecular Biology, Guangdong Medical College, Zhanjiang, Guangdong 524023, P.R. China

**Keywords:** metformin, cisplatin-resistant lung cancer cells, p38 MAPK

## Abstract

Metformin (Met) has been widely used in hypoglycemic therapy, and it is also able to reduce the incidence of tumors and tumor-related mortality. The present study investigated whether Met could inhibit the proliferation of lung cancer cells and enhance the sensitivity of a cisplatin-resistant lung cancer A549/CDDP cell line to cisplatin. It was found that Met treatment inhibited the proliferation of different lung cancer cells. Met inhibited the cell cycle of the A549/CDDP cells and induced apoptosis. Upon Met treatment, the A549/CDDP cells were arrested at the G_1_ phase. The apoptosis of the A549/CDDP cells was confirmed by the appearance of apoptotic bodies in cells stained with Hoechst 33258, and by the cleavage of BH3 interacting-domain death agonist and poly (ADP-ribose) polymerase. Furthermore, results showed that the phosphorylation level of p38 mitogen-activated protein kinase (MAPK) was increased after Met treatment. The p38 MAPK inhibitor, SB203580, significantly blocked Met-induced apoptosis in the A549/CDDP cells. It was further demonstrated that Met could enhance the sensitivity of the A549/CDDP cells to cisplatin. In summary, the present study identified Met as a drug sensitizer that could improve the treatment effect of cisplatin in cisplatin-resistant lung cancers.

## Introduction

Lung cancer is one of the most common malignant tumors in the world. Clinical trials have shown that cisplatin-based chemotherapy can significantly improve the survival rate of inoperable non-small cell lung cancer patients (1), but resistance to cisplatin limits its wider use in clinical applications. At present, the overall five-year survival rate of lung cancer patients is <15% (2). Cisplatin resistance is the main reason for the failure of cancer chemotherapy, which contributes to the difficulty in providing a lung cancer cure and a poor long-term survival rate. Therefore, finding an effective method or medicine to reverse cisplatin resistance is a reasonable strategy to solve the drug resistance problem in lung cancer. To this end, the study of the mechanism of cisplatin resistance in lung cancer is key. During chemotherapy, cisplatin takes effect through the induction of lung cancer cell apoptosis. The resistance to cisplatin-induced cell apoptosis is considered as one of the important mechanisms of drug resistance in lung cancer (3).

Metformin (Met) has been widely used in hypoglycemic therapy in patients with type 2 diabetes (4). It has been shown in clinical observations that Met could reduce the incidence of tumors and the mortality of cancer patients (5). In recent years, numerous studies have confirmed that Met has anti-cancer effects (5–10). However the mechanism with regard to how Met affects the proliferation of lung cancer is not clear. It is also unclear whether Met could increase the sensitivity of cancer cells to cisplatin treatment. The present study investigated the effect of Met on the proliferation of different lung cancer cells and its effect on the cisplatin resistance. The study also provides a discussion on the mechanism of Met action, and conducts a preliminary evaluation of Met as an anticancer drug.

## Materials and methods

### Cell culture

The human lung cancer A549, cisplatin-resistant lung cancer A549/CDDP, SPCA and H23 cell lines were kept in the Institute of Biochemistry and Molecular Biology in Guangdong Medical College (Zhanjiang, Guangdong, China). The cell lines were cultured in the Dulbecco’s modified Eagle’s medium (Gibco BRL, Carlsbad, CA, USA) supplemented with 10% fetal bovine serum (Sijiqing Laboratories, Hangzhou, Zhejiang, China), 100 μg/ml penicillin and 100 μg/ml streptomycin. The cells were incubated at 37°C in a humidified atmosphere with 5% CO_2_.

### Reagents

Met and MTT were purchased from Sigma-Aldrich (St. Louis, MO, USA). Rabbit and goat polyclonal antibodies against cytochrome *c*, BH3 interacting-domain death agonist (Bid), poly (ADP-ribose) polymerase (PARP), caspase-3, caspase-8, β-actin and SB203580 were purchased from Santa Cruz Biotechnology, Inc. (Santa Cruz, CA, USA). The antibodies against phosphorylated forms of p38 MAPK-Thr180/Tyr182 and against p38 MAPK were purchased from Cell Signaling Technology (Beverly, MA, USA).

### Cell viability assay

The A549, A549/CDDP, SPCA and H23 cells were treated with different concentrations of Met (0, 0.5, 1, 2,4 and 8 mmol/l) for various times (24, 48 and 72 h) and the cell viability was determined by MTT assay, as previously described (11).

### Analysis of cell apoptosis by fluorescence staining

The A549/CDDP cells were treated with 4 mM Met for the different time periods at 37°C. Priot to the cells being examined under a fluorescence microscope, they were incubated in Hoechst 33258 (10 mg/l) solution at 37°C for 20 min. Apoptosis was evaluated by the uptake of Hoechst 33342 (12). The apoptotic index was determined by dividing the number of apoptotic nuclei by the number of total nuclei.

### Analysis of the cell cycle by flow cytometry

The cells were collected by centrifugation at 1,000 × g following treatment with 4 mM Met for the different time periods at 37°C. The cells were then washed twice with phosphate-buffered saline (PBS) and fixed with ice-cold 70% ethanol overnight. Prior to the flow cytometry analysis for cell cycle distribution, the fixed cells were washed once with PBS (Sigma-Aldrich) and incubated with 100 μg/ml propidium iodide (Sigma-Aldrich) plus 200 μg/ml RNase (Sigma-Aldrich).

### Treatment with p38 MAPK inhibitors

The ells were preincubated with the MAPK inhibitor, SB203580 (10 μM), for 2 h and then treated with Met (4 mM) for 24 h. Cell viability was determined using an MTT assay and the protein levels were measured by western blotting.

### Western blotting

The cells were lysed with cell lysis buffer (pH 8.0) containing 50 mM Tris-HCl (Sigma-Aldrich), 150 mM NaCl (Guangzhou Chemical Reagent Factory, Guangzhou, China), 5 mM EDTA (Guangzhou Chemical Reagent Factory), 1% NP40 (Sigma-Aldrich), 0.05% phenylmethanesulfonyl fluoride (Sigma-Aldrich), 2 μg/ml aprotinin (Sigma-Aldrich) and 2 μg/ml leupeptin (Sigma-Aldrich). The protein levels were determined by western blotting, as described previously (4).

### Treatment of A549/CDDP cells with Met and cisplatin

The A549/CDDP cells were treated with the different concentrations of Met and cisplatin for 24 h at 37°C. Cell viability was determined using an MTT assay.

### Statistical analysis

Results are presented as the mean ± standard deviation. Statistical analysis was performed using a one-way analysis of variance with a least significant difference test. P<0.05 was considered to indicate a statistically significant difference.

## Results

### Effect of Met on the growth of different lung cancer cell lines

**Figure 1:**
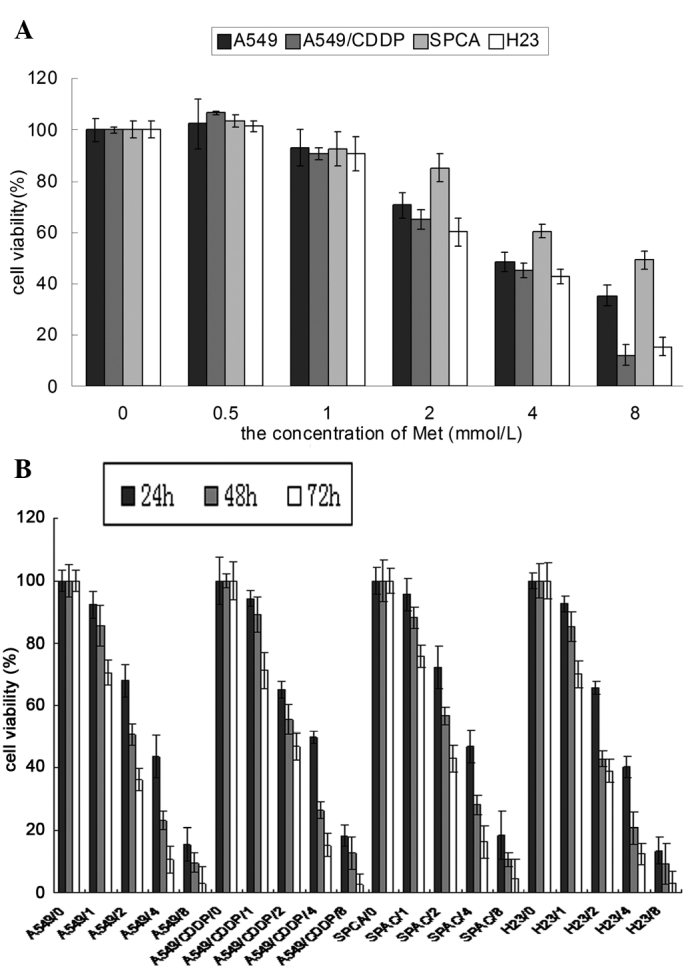
Effect of Met on the growth of the different lung cancer cell lines. (A) Different lung cancer cells were treated with varying concentrations of Met for 24 h. The data are presented as the mean ± standard deviation (n=4). (B) Different lung cancer cells were treated with varying concentrations of Met for a range of times. The data are presented as the mean ±standard deviation (n=4). Met, metformin.

Fig. 1 showed that Met inhibited the proliferation of the lung cancer cells in a concentration- and time-dependent manner. The proliferation of the lung cancer cells was significantly inhibited by 24 h of Met treatment at concentrations of 2–8 mmol/l (P<0.05). The survival of the lung cancer cells decreased significantly compared with the control group following 48 h of treatment with 1 mmol/l Met (P<0.05). It was also shown that the response of the A549/CDDP cells was similar to its parent cell line, A549.

### Met inhibits the A549/CDDP cell cycle and induces apoptosis

**Figure 2:**
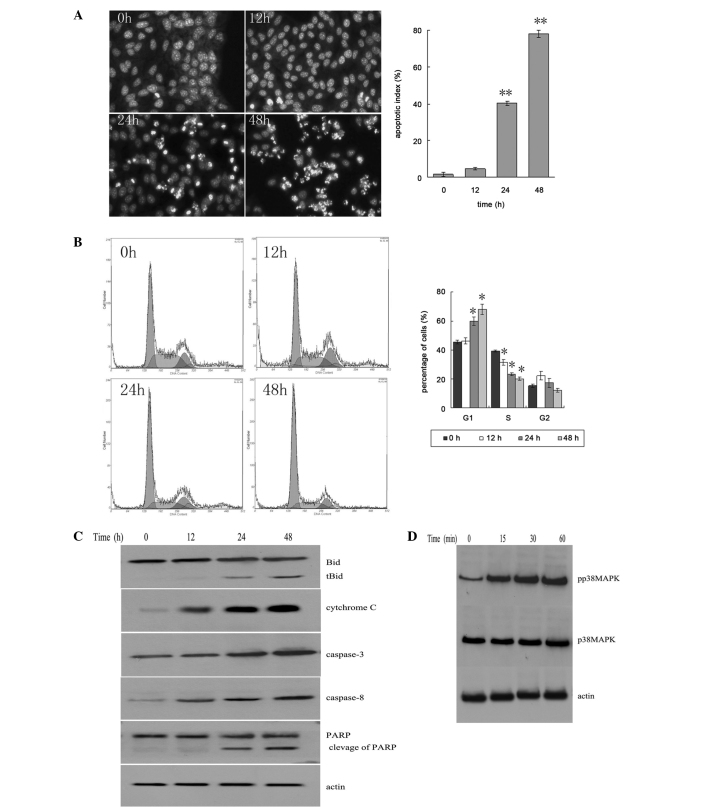
Met inhibits the A549/CDDP cell cycle and induces apoptosis. (A) Analysis of cell apoptosis by fluorescence staining. Images of the A549/CDDP cells following treatment with Met for different times. Nuclei were stained with Hoechst 33258 (magnification, ×200). The apoptotic index was determined by calculating the percentage of the apoptotic nuclei as a portion of the total nuclei. Data are presented as the mean ± standard deviation (n=4). ^*^P<0.05, ^**^P<0.01 vs. 0 h group, respectively. (B) Flow cytometric analysis of cell cycle distribution after different periods of Met treatment. Data are presented as the mean ± standard deviation (n=3). ^*^P<0.05, ^**^P<0.01 vs. 0 h group, respectively. (C) The expression levels of proteins as determined by western blotting. (D) The level of p38 MAPK as determined by western blotting. Met, metformin; MAPK, mitogen-activated protein kinase; PARP, poly (ADP-ribose) polymerase; Bid, BH3 interacting-domain death agonist.

The results showed that a large number of apoptotic cells appeared following 24–48 h of Met treatment (Fig. 2A). This was demonstrated by the apoptotic bodies in the cells stained with Hoechst 33258 (Fig. 2B). With Met treatment, the number of cells in the G_1_ phase was increased, and the number of cells in the S phase and G_2_ phase was decreased (Fig. 2C).

### Met induces the cleavage of Bid and PARP

The fragments of Bid and PARP increased with the increase in incubation time. The levels of caspase-3, caspase-8 and cytochrome *c* increased in the cytosol (Fig. 2C). It was shown that the phosphorylation level of p38 MAPK was increased following 15 min of Met treatment, indicating that Met could activate the p38 MAPK pathway (Fig. 2D).

### Reduction of Met-induced cell apoptosis by inhibition of p38 MAPK activity

**Figure 3:**
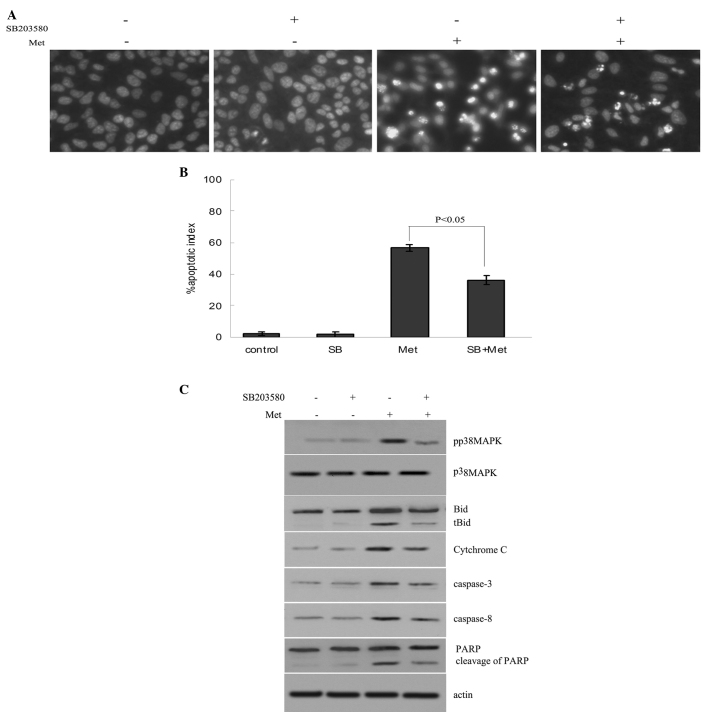
A549/CDDP cells were pre-incubated with 10 μM SB203580 for 2 h, followed by incubation with 4 mM Met for 24 h. (A) Cells were stained with Hoechst 33258 (x200). (B) The apoptotic index was determined by calculating the percentage of the apoptotic nuclei as a proportion of the total nuclei. Data are presented as the mean ± standard deviation (n=4). (C) The expression levels of proteins as determined by western blotting. Met, metformin; MAPK, mitogen-activated protein kinase; PARP, poly (ADP-ribose) polymerase; Bid, BH3 interacting-domain death agonist; SB, SB203580 p38 MAPK inhibitor.

The A549/CDDP cells were preincubated with 10 μM SB203580 (a p38 MAPK inhibitor) for 2 h, and then treated with 4 mM Met for 24 h. The results showed that Met activated p38 MAPK and induced the apoptosis of the A549/CDDP cells. Met-induced apoptosis was inhibited by SB203580 (Fig. 3A and B). The data also showed that SB203580 decreased the levels of Bid and PARP cleavage fragments, and the caspase-3, caspase-8 and cytosolic cytochrome *c* levels (Fig. 3C).

### Met enhances the sensitivity of A549/CDDP cells to cisplatin

Consistent with our previous study (12), the results showed that the IC_50_ values of cisplatin for the A549 cells and A549/CDDP cells were 2.36±0.10 and 30.27±1.50 μmol/l, respectively. The difference between the cisplatin-resistant and parent cells was 12.8-fold.

**Figure 4:**
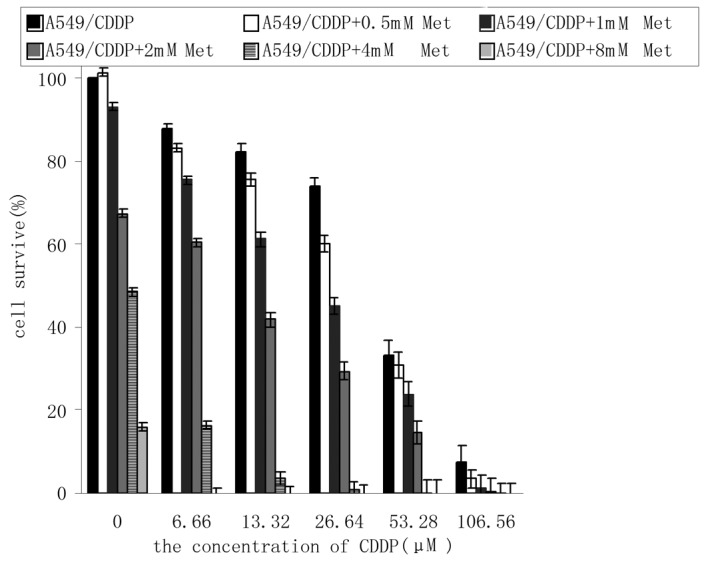
Met enhances the sensitivity of A549/CDDP cells to cisplatin (CDDP). The A549/CDDP cells were treated with different concentrations of Met and cisplatin for 24 h. The data are presented as the mean ± standard deviation (n=4). Met, metformin.

In this experiment, it was shown that the IC_50_ of cisplatin for the A549/CDDP cells was 32.18±1.15 μmol/ in the absence of Met, while the IC_50_ values decreased to 27.45±2.38, 19.56±1.60, 9.65±1.38, 0.06±0.07 and 0.00±0.05 μmol/l respectively in the presence of 0.5, 1, 2, 4 and 8 mmol/l Met (P<0.05 vs. control) (Fig. 4). Met evidently increased the cytotoxicity of cisplatin.

## Discussion

Every year, 1,200,000 patients are diagnosed with lung cancer, and ~25% of all cancer mortalities are patients with lung cancer, representing a large threat to human health (13,14). Chemotherapy and radiotherapy are the major methods for the treatment of patients with lung cancer. However, chemotherapy drugs have toxicity to normal tissue, and may cause serious side-effects in the clinic. Furthermore, the chemotherapy and radiotherapy drugs are expensive. Therefore it is of great import to search for effective antitumor drugs with a low toxicity and cost, which can enhance the effect of chemotherapy and improve the prognosis of lung cancer patients.

Met is an effective antihyperglycemic agent, which has been widely used in the treatment of patients with type 2 diabetes for decades. Certain studies have shown that Met not only inhibits breast cancer growth in HER-2/neu transgenic mice (6), but that it also inhibits pancreatic cancer induced by feeding of a high-fat diet in hamsters (7). Clinical data has also revealed that Met has anti-tumor activity. Evans *et al* (5) reported that the risk for tumorigenesis in type 2 diabetes patients administered Met treatment is 23% lower than those subjected to sulfonylurea treatment. Furthermore, after following 10,309 patients with newly diagnosed type 2 diabetes for approximately five years, Bowker *et al* (8) concluded that treatment with Met could be associated with lower mortality for malignancies in comparison with sulfonylureas in type 2 diabetes patients. Buzzai *et al* (9) demonstrated that Met has toxicity towards p53-mutated colorectal cancer cells. Ben Sahra *et al* (10) showed that Met inhibits the proliferation of prostate cancer cells, but not normal prostate epithelial cells. The present study data also showed that Met inhibited lung cancer cell growth and proliferation in a time- and concentration-dependent manner, which is consistent with previous results (5–9). In addition, the A549/CDDP cells were sensitive to Met. These findings suggest that Met may have a wide range of antitumor effects.

Cell proliferation is mainly affected by two factors, the cell cycle and apoptosis. Ben Sahra *et al* (10) showed that Met suppresses prostate cancer cell proliferation by arresting the cell cycle in the G_1_ phase. However, it has no evident effect on cell apoptosis. However the present results demonstrated that Met not only induced A549/CDDP cell arrest at the G_1_ phase, but that it also promoted cell apoptosis.

Cisplatin is the most commonly used non-specific anticancer drug, which binds to tumor cell DNA and interferes with its function (15). The present study found no significant difference in the sensitivity to Met between the A549/CDDP cells and its parent cell line, A549. Furthermore, Met enhanced the sensitivity of the A549/CDDP cells to cisplatin when the cells were treated with Met and cisplatin together. The results suggest that Met may be used to enhance the cisplatin toxicity in cisplatin-resistant lung cancer cells and to improve the effectiveness of cisplatin-based chemotherapy in lung cancer patients.

Cisplatin has been used to treat tumors based on its ability to induce the apoptosis of tumor cells. One important mechanism of drug resistance in lung cancers is the tolerance of cancer cells to cisplatin-induced apoptosis. Apoptosis pathways include mainly the death receptor, mitochondrial and endoplasmic reticulum stress pathways. p38 MAPK and caspase family proteins play key roles in these apoptotic signal pathways.

The activation of p38 MAPK can induce apoptosis in various tumor cells. Jiang *et al* (16) reported that selenite activated p38 MAPK activity and promoted apoptosis in Jurkat cells. Inhibiting the p38 MAPK pathway can effectively reduce the release of cytochrome *c* and inhibit Caspase-3 activation and PARP cleavage, resulting in the decrease of apoptosis cells (17). Mandal *et al* (18) showed that the p38 MAPK pathway is indispensable in the apoptosis of leukemia cells induced by Withaferin A. The p38 MAPK signaling pathway plays a key role in promoting the apoptosis of cells, a process associated with the activation of p53 and caspases, and the translocation of Bax (19,20). A study by Khan *et al* (21) demonstrated that the activation of the p38 MAPK pathway can induce the activation of caspases, and that the inhibition of p38 MAPK can reduce the expression levels of caspase activated protein. The study suggested that p38 MAPK is an upstream regulator of the caspase-dependent signal transduction pathway of apoptosis (21). The present study showed that Met induced the phosphorylation of the p38 MAPK protein and increased the expression of caspase-8 and caspase-3, along with the cleavage of Bid and PARP, and cytochrome *c* release in the cytoplasm. These results suggest that Met may promote the expression of caspase-8 by activating p38 MAPK phosphorylation. Bid was cleaved by activated caspase-8 to produce tBid, which migrated to the mitochondrial membrane, resulting in the change of mitochondrial membrane permeability, cytochrome *c* release, caspase-3 activation and cleavage of PARP to promote apoptosis. Inhibition of p38 MAPK significantly reduced the Met-induced apoptosis of the A549/CDDP cells. These results indicate that the p38 MAPK-caspase pathway may be involved in the regulation of A549/CDDP cell apoptosis induced by Met. Although the inhibition of the p38 MAPK signal pathway reduced the level of Met-induced A549/CDDP cell apoptosis, it did not completely inhibit the apoptosis of the A549/CDDP cells. This result suggests that other factors may participate in the regulation of A549/CDDP cell apoptosis induced by Met.

Results of the present study support the hypothesis that the proliferation of A549/CDDP cells can be inhibited by Met, which may be used as an adjuvant therapy to improve the clinical treatment effect of cisplatin in cisplatin-resistant lung cancers. Met is widely used as an insulin sensitizer and has shown few toxic side-effects in a number of years of clinical practice. Further studies are required to explore the other clinical applications of Met.

## Abbreviations

Met: metformin
MAPK: mitogen-activated protein kinase
